# The Enigmatic CA2: Exploring the Understudied Region of the Hippocampus and Its Involvement in Parkinson’s Disease

**DOI:** 10.3390/biomedicines11071996

**Published:** 2023-07-14

**Authors:** Fang Zhao, Thomas Behnisch

**Affiliations:** State Key Laboratory of Medical Neurobiology and MOE Frontiers Center for Brain Science, Institutes of Brain Science, Fudan University, Shanghai 200032, China; 19111520036@fudan.edu.cn

**Keywords:** memory, hippocampus, CA2, Parkinson’s disease, social cognitive deficits

## Abstract

Parkinson’s disease (PD) is a neurodegenerative disease that affects both motor and non-motor functions. Although motor impairment is a prominent clinical sign of PD, additional neurological symptoms may also occur, particularly in the preclinical and prodromal stages. Among these symptoms, social cognitive impairment is common and detrimental. This article aims to review non-motor symptoms in PD patients, focusing on social cognitive deficits. It also examines the specific characteristics of the CA2 region and its involvement in social behavior, highlighting recent advances and perspectives. Additionally, this review provides critical insights into and analysis of research conducted in rodents and humans, which may help improve the understanding of the current status of putative therapeutic strategies for social cognitive dysfunction in PD and potential avenues related to the function of the hippocampal CA2 region.

## 1. Introduction

Parkinson’s disease (PD) is a debilitating neurodegenerative disorder characterized by the loss of dopaminergic neurons in the pars compacta of the substantia nigra, a loss that is unfortunately irreversible [1]. Studies have described many non-motor symptoms that appear in the early stages of PD [2,3], in particular, social cognitive decline such as perception, language, and decision-making [4], as well as temporal-order memory deficits [5]. Interestingly, the hippocampal CA2 region, with its unique properties, has attracted the attention of scientists due to its potential association with social cognitive functions. In this review, we first briefly describe the unique properties of the CA2 region and its putative social functions. We then review the social cognitive deficits found in PD patients and examine the evidence linking them to the CA2 region. In addition, we compare findings from rodent and human studies. Finally, we summarize potential therapeutic drugs that show promise in attenuating social cognitive dysfunction in Parkinson’s disease (PD), including those that target the CA2 region.

## 2. The Hippocampal Formation and the Unique Properties of the CA2 Region

The hippocampal formation (HF) is located within the medial temporal lobe in all mammalian species, close to the adjacent cerebral cortex, enabling its many crucial connections to various cortical regions. HF is a critical functional unit that contributes significantly to many vital cognitive processes in both humans and animals, including learning and memory [6], fear processing [7], spatial orientation [8], and social behavior [9]. HF includes the cornu ammonis (CA) and the dentate gyrus areas. Ramón and Cajal divided the CA into two parts: the superior region, composed predominantly of small-body neurons, and the inferior region, composed of larger vertebral-body neurons. This division was later refined by Rafael Lorente de Nó, who identified four subregions within the CA area: CA1, CA2, CA3, and CA4. He observed that the neurons in CA2 and CA3 were larger than those in CA1 and that the CA2 subregion did not receive mossy fiber projections from the DG but instead received inputs via the Schaffer collateral fibers originating in CA3 [10]. However, Dudek et al. determined that the extent to which mossy fibers project into CA2 and synapse onto CA2 pyramidal neurons is species dependent [11]. The nomenclature and function have long been subjects of controversy, with debate over whether it is a distinct or transitional region between CA1 and CA3. However, current research suggests that CA2 possesses a unique biological structure [12], which requires further investigation in humans [13]. Interestingly, researchers have also indicated that CA2 may be proportionally larger in primates than in rodents [11].

CA2 exhibits unique morphological features, including a more loosely packed stratum pyramidal in comparison to CA1. In addition, pyramidal neurons in CA2 have an oval and dense soma, which is the largest among excitatory neurons within CA regions. Neurons in CA2 are also characterized by a hyperpolarized resting membrane potential and display specific action potential firing patterns [14]. Furthermore, the afferent and efferent connections of the CA2 field have distinct origins and terminations compared to other regions of the hippocampal formation. For instance, an optogenetic study demonstrated functional monosynaptic inputs from the DG via longitudinal projections to the CA2 area [15]. Other data suggest that CA2 neurons have more extensive functional synaptic connections with the deep area of CA1 than with the superficial layer [15] and that they also exhibit stronger innervation to CA1 than to CA3 [14]. However, the degree of synaptic connectivity between layer III EC afferents and distant branches of CA2 neurons may vary between species. In particular, fibers originating from layer III EC neurons and traversing the stratum lacunosum-moleculare in the CA1 region play a role in this variation [14].

Recently, CA2 has been shown to play a central role in social behavior. Molecular markers such as Purkinje cell protein 4 (PCP4), a regulator of G protein signaling 14 protein (RGS14), and striatum-enriched protein–tyrosine phosphatase (STEP), help to identify the specific population of neurons in the CA2 region [11,12,15,16,17,18]. Lee et al. demonstrated that RGS14 deletion imparts a substantial capacity for SC-CA2 synapse, whereas wild-type CA2 neurons exhibited little LTP [16]. Researchers discovered a loss of inhibitory neurons in CA2 in a neuropsychiatric disorder-like mouse model. These mice exhibited impaired social cognition and reduced synaptic plasticity in CA2, which may be related to the loss of PV+ interneurons [19]. In addition, CA2 activates a disinhibitory circuit from the lateral septum to the ventromedial hypothalamus (LS-VMHv1), which is modulated by the signaling pathway of arginine vasopressin (AVP), a hormone and neurotransmitter, to promote social aggression [20]. Dysfunctions in this signaling pathway have also been associated with neuropsychiatric disorders such as depression, anxiety, and autism spectrum disorders. Furthermore, researchers have speculated that the CA2 region is crucial for the formation and retrieval of memories related to social encounters [21]. Although arginine vasopressin receptor 1B (AVPR1B) mRNA is highly expressed in CA2 pyramidal neurons in both humans and rodents [12,21], one study demonstrated that AVPR1B -deficient mice were unable to recognize other mice in the “social novelty test” and also showed impaired chronological-order memory [22].

Another test showed that AVPR1B knockout mice could not discriminate the object they explored and recognize its location like the control group [23]. AVPR1B deficiency in CA2 impaired social memory enhancement [24]. In addition to AVPR1B, oxytocin receptors, another social neuropeptide receptor, are also highly expressed in CA2 [25,26]. In addition, genetic evidence suggests that CA2 injury impairs social recognition in mice [22]. Interestingly, the CA2 area of the hippocampus is the only region that receives vasopressinergic input from both the paraventricular nuclei of the hypothalamus and the supramammillary nuclei (SuM)—a critical factor in the regulation of social cognitive behaviors [27,28,29,30]. Interestingly, terminals belonging to the SuM have been found to express substance P [11], which plays a central role in PD. Furthermore, research suggests that these particular SuM afferents expressing substance P specifically target CA2 in rats and have the ability to influence plasticity in pyramidal neurons located in CA2 [31]. The SuM-to-CA2 projection has also been reported in monkeys and humans and occurs during early embryonic development [32]. The reason for enhanced social performance may involve the circuit from dorsal CA2 to ventral CA1 [33], spike timing-dependent plasticity in CA2 [34], the negative regulatory role of CA2 in hippocampal sharp-wave ripples [35,36], and the distinct dendritic properties of CA2 compared to CA1 [37]. In addition, mineralocorticoid receptors (MRs) have been shown to facilitate CA2-dependent behaviors [38].

In summary, CA2 pyramidal neurons possess numerous distinctive morphological, physiological, and synaptic characteristics, as well as intrinsic and extrinsic connections that distinguish them from other CA regions (see Table 1), and more DEGs that are unique for CA2 regions have been described [11]. Despite the identification of several molecular markers in this area by current studies, our understanding of its functional properties, including its unique physiology, signaling and resilience, and behavioral role, particularly in synaptic plasticity and PD, remains limited.

## 3. Dopamine and Social Behavior in Parkinson’s Disease

PD is an irreversible neurodegenerative disease [1] characterized by the loss of dopaminergic neurons in the pars compacta of the substantia nigra. Dopamine replacement therapy is the primary treatment for PD, aiming to alleviate symptoms and improve the quality of life [55]. However, the role of dopamine extends beyond motor function. It also plays a crucial role in memory formation, particularly in the hippocampus. Research has shown that dopamine activity in the hippocampus is associated with cognitive function, such as social recognition memory in mice [56] and stimulus generalization in humans [57]. Furthermore, dopamine is critical for social abilities, as evidenced by studies demonstrating that dopamine receptor antagonists can reduce social play in rats [58,59]. Interestingly, there is a complex relationship between stress, social cognition, and the dopamine system [60,61]. Research has shown that the dopamine D1 receptor in the lateral nucleus of the cerebellum (LCN) is involved in spatial navigation and the formation of social recognition memory in both mice and humans [62]. In animal models of autism, an intriguing correlation has been observed between reduced dopamine neuron activity and impaired social preference [63]. Additionally, dopamine neurons in the dorsal raphe nucleus (DRN) have been found to be involved in social isolation and can mediate a “loneliness-like” state in mice [64]. Recently, a study proposed that the dopaminergic metabolic pathway was specifically disrupted in the hippocampus and prefrontal cortex (PFC) of chronically socially defeated and stressed mice [65]. Furthermore, dopamine transporter (DAT) proteins were found to be enriched in patients with social anxiety disorder [66]. Interestingly, a recent study discovered specific cells with firing patterns similar to dopamine D1 receptor-like neurons in the medial prefrontal cortex (mPFC) that are necessary and sufficient for social memory in mice [67]. However, the role of dopamine itself in CA2 function, particularly in the context of PD, requires further investigation.

## 4. Complex Changes in the Social Behavior of People with Parkinson’s Disease

Indeed, motor, autonomic, and cognitive impairments are commonly observed in PD [68]. Studies have reported social cognitive decline, including perception, language, and decision-making [4], as well as temporal-order memory deficits [5], in the early stages of PD. PD patients typically exhibit rigidity, tremor, and bradykinesia as classical motor symptoms. Notably, PD patients not only experience multiple motor symptoms but also encounter cognitive deficits [69] and other neuropsychological symptoms, such as depression and anxiety [70,71]. Cognitive impairment can also affect executive function, which encompasses higher-level cognitive processes such as decision-making and problem-solving. Moreover, autonomic neurological deficits, including insomnia, constipation, and lower urinary tract symptoms (LUTS), can exacerbate social deficits. Typically, individuals with motor dysfunction alone but normal cognition are diagnosed with PD, whereas those with motor dysfunction and dementia are classified as having PD dementia (PDD) or PD with mild cognitive impairment (PD-MCI). Up to 25% of people with PD have MCI and are at increased risk of developing PDD [72]. It is estimated that 10%–80% of people with PD are likely to develop PDD [73]. Research suggests that social cognitive deficits are present in the early stages of PD and can worsen as the disease progresses [4]. Social cognition includes the perception of facial expressions, personality, behavior, and relationships, and it involves inferring and judging information from social interactions [74]. PD patients exhibit social cognitive impairments in several aspects, including decision-making, emotion, recognition, and empathy. The theory of mind (ToM), which includes affective and cognitive aspects, is the most representative theory of social cognition. It refers to the ability of individuals to understand their own and others’ psychological states and to predict and explain the behavior of others [75]. Studies demonstrate that people with PD score lower on ToM assessments compared to healthy individuals. This suggests that both affective [76,77] and cognitive aspects of ToM are impaired and that these impairments worsen as the disease progresses [78]. Visuospatial abilities contribute significantly to ToM [77]. In addition, research suggests that these impairments are separate from other PD symptoms, such as cognitive function, depression, and motor impairments [79]. Poor performance on advanced ToM tasks in PD patients is associated with executive function deficits [80].

Patients with PD have impaired decision-making, as studies have shown that they have difficulty learning from feedback and making optimal decisions, possibly due to dopamine depletion in the basal ganglia [81]. PD patients have difficulty making appropriate judgments in risky situations [81,82,83] and exhibit impaired social communication skills [84]. In addition, moral decision-making in PD patients differs from that of healthy individuals and is based on ToM [85] rather than stress [86]. Patients with PD and mild cognitive impairment show difficulties with problem-solving [87], suggesting a possible link between social cognitive deficits and executive dysfunction [88,89]. PD patients also have difficulty recognizing negative emotions and showing empathy [90,91,92,93,94]. These deficits may be related to dopaminergic depletion in the limbic system and prefrontal cortex [95,96]. However, there are few studies that showed that dopamine replacement therapy failed to abolish ToM deficits, suggesting that the dopaminergic mechanism may not be involved [97,98].

The intricate alterations in social behavior observed in individuals with PD present a challenge in identifying precise molecular mechanisms or neural circuits, including the hippocampal CA2 region, that contribute to these changes. Further research is necessary to establish a correlation between CA2 functionality and specific social behavioral variations observed in PD patients. Nonetheless, a few studies have indicated a potential involvement of CA2 in human social behavior [99,100,101]. Furthermore, there is evidence that some other diseases with social cognitive deficits, such as schizophrenia, showed apparent changes in human CA2. This collective body of research highlights the significant role of the human hippocampal formation in social cognition [102,103,104,105,106], as summarized in Table 2.

## 5. Exploring the Role of CA2 in Parkinson’s Disease

As the classical view of PD expands beyond its traditional characterization as a purely motor disorder, it becomes increasingly important to delve into the contributions of non-motor brain regions. Among these regions, the hippocampal formation has been widely studied, and findings indicate that the hippocampus may possess compensatory mechanisms that help mitigate cognitive impairments associated with PD [120]. In parallel, explorations of the CA2 region within the hippocampus have illuminated a noteworthy association between the frequency of cortical Lewy bodies and the extent of neuritic degeneration [121].

The presence of α-synuclein in brainstem nuclei serves as a defining feature of PD and is closely linked to motor impairment. However, it is worth noting that α-synuclein also accumulates in the cerebral cortex and hippocampus, where it plays a crucial role in the development of cognitive abilities [46,47]. This accumulation has been shown to have detrimental effects on synapses by activating neuronal extrasynaptic NMDA receptors (eNMDARs) [49]. Interestingly, within the hippocampus, α-synuclein is particularly enriched in the CA2 region [47]. Notably, postmortem studies have revealed a strong correlation between α-synuclein accumulation in CA2 and dementia in PD and PDD patients [50,51]. In this context, phosphorylated human S129-α-synuclein, which constitutes the majority of α-synuclein in Lewy bodies, has been found to predominantly accumulate in CA2 compared to other regions of the hippocampus in a transgenic PD-like mouse model [122]. Interestingly, in stage III of PD in humans, α-synuclein expression was significantly elevated only in CA2, whereas other hippocampal areas showed lower levels. The highest levels of α-synuclein in CA2 were observed in stages IV and V of PD [51]. However, similar findings were not observed in A53T mice. Notably, another study reported the accumulation of α-synuclein in the stratum lacunosum-moleculare (SLM) of CA2 in mice, which was associated with innervation from the entorhinal cortex (EC) [52]. Furthermore, some researchers have proposed that this type of pathology in CA2 may be related to cholinergic dysfunction [53,54].

**Figure 1:**
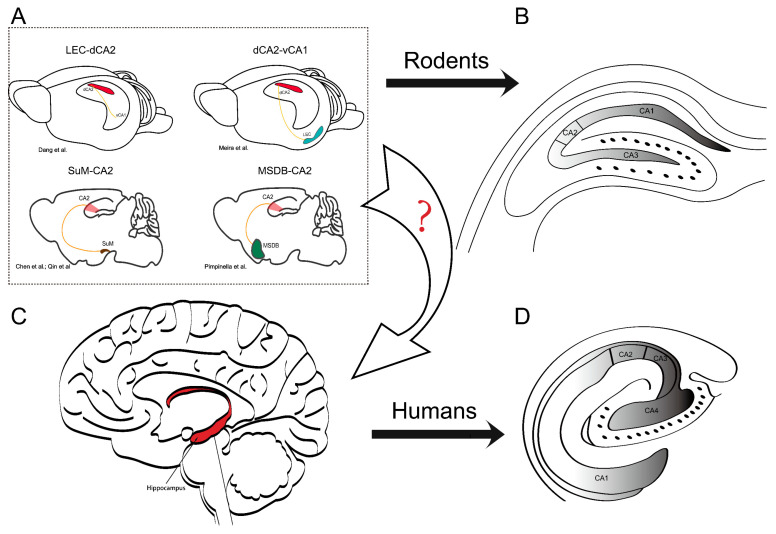
**Interbrain connectivity to CA2 and differences in the localization and orientation of the hippocampal formation in rodents and humans.** (**A**) The sketch shows the hippocampal formation and the localization of basic brain-wide connectivity associated with afferents to CA2. The figure highlights four brains with distinct connectivity patterns. In addition, recent studies highlight the need to clarify the organization of these specific circuits in humans: lateral entorhinal cortex (LEC)-CA2 [127], dCA2-vCA1 (d: dorsal, v: ventral) [33], supramammillary area (SuM)-CA2 [29,30], and medial septum–diagonal band of the Broca complex (MSDB)-CA2 [128]. These circuits play a critical role in hippocampal function, and understanding their organization (arrow with “?”) in humans may have implications for various neurological and psychiatric disorders. (**B**) The CA regions in a rodent hippocampal slice. (**C**) Human brain and location of the hippocampus. The red area indicates the hippocampal formation. (**D**) The CA regions in the human hippocampal formation appear to be mirrored with respect to the orientation in the rodent. The figure panels (**B**,**D**) from the reference [129] were modified to emphasize the different orientation of the hippocampal formation in humans and rodents.

However, a recent study conducted in mice demonstrated that the infusion of α-synuclein fibrils into the CA2–CA3 region did not result in behavioral deficits or cell loss, despite the presence of α-synucleinopathy in this area [45]. However, the model in this study mimics the early stage of PD, suggesting the need to design experiments that address the later stages of PD, when cognitive impairments become more prevalent. On the other hand, it may illustrate the protective role of CA2 in the early stage of this disease. CA2 may not only manifest a unique feature in physiology but also show a different fate in response to different diseases compared to other CA regions. For instance, studies investigating temporal lobe epilepsy in rats [123] and brain injury in humans [124] have consistently shown that pyramidal neurons in CA2 display remarkable resilience to cell death. Additionally, a study focusing on hippocampal sclerosis indicated that neuronal loss in CA2 was less severe compared to other areas of the hippocampus [125]. This suggests that CA2 may possess a distinct resistance to neuronal impairment, possibly due to its robust calcium-handling capabilities, which reduce its vulnerability to cytotoxic events [126]. Furthermore, recent investigations have underscored the importance of considering specific circuits associated with CA2: SuM-CA2 [29,30], lateral entorhinal cortex (LEC)-CA2 [127] and the medial septum-diagonal band of Broca complex (MSDB)-CA2 [128]. However, it remains essential to gain a comprehensive understanding of how these circuits are organized in humans (Figure 1A).

The findings suggest that social cognitive impairments are present in both individuals with PD and animal models of the disease, potentially linked to CA2 function. However, the extent to which CA2 contributes to these cognitive deficits, such as social interaction and temporal ordering, remains uncertain. Furthermore, considering the structural disparities between rodents and humans [129], it is imperative to investigate whether humans exhibit similar functional properties and interbrain connectivity to CA2 as observed in rodents (Figure 1B,C).

The treatment of PD involves various medications, but there is a notable lack of effective options for addressing non-motor symptoms, particularly social disorders. Therefore, it is crucial to identify treatments that can specifically target and alleviate these symptoms without exacerbating motor impairments. In this context, understanding the therapeutic potential of the CA2 region becomes highly relevant. Existing research has examined the effects of specific drugs on the CA2 region (see Table 3). For example, vasopressin, commonly used for diabetes insipidus, has been found to inhibit long-term potentiation (LTP) in the CA2 [130]. Furthermore, dantrolene, a medication for malignant hyperthermia, and ketamine, an anesthetic and antidepressant, have shown promise in rescuing CA2 apoptosis following electroconvulsive seizures (ESC) [131]. Studies in rats have shown that antipsychotics such as haloperidol, clozapine, and olanzapine can reduce the expression of the neuronal glutamate transporter EAAT3 [132] and NMDA receptors in CA2 [133]. In addition, oxytocin, known to improve social cognitive deficits in autism, may exert its effects via somatostatin interneurons in the DG and CA2/CA3 regions [41]. Notably, a human study suggested that long-term treatment with L-DOPA can restore the CA2 volume in patients with PD [134].

In summary, several medications hold potential for improving social memory, enhancing sociability, and reducing social anxiety. Among these is oxytocin, a hormone renowned for its role in promoting social bonding and trust. Studies have suggested that intranasal administration of oxytocin may improve social memory, increase sociability, and reduce social anxiety. Another class of medications, selective serotonin reuptake inhibitors (SSRIs), commonly prescribed as a treatment for depression and anxiety disorders, may also have a positive effect on social anxiety and social functioning. In addition, certain drugs that act as NMDA receptor antagonists, such as ketamine, have shown potential for improving sociability and reducing social anxiety, particularly in individuals with conditions such as autism spectrum disorder [131].

Furthermore, the review of existing literature uncovers the potential impact of specific traditional Chinese medicines (TCMs) on the CA2 region. For example, administration of CS 4-O-sulfation increases the presence of perineuronal networks (PNNs) and excitatory–inhibitory synapses in CA2 [135]. Another chemical compound, dihydroartemisinin, derived from TCM artemisinin, has exhibited protective effects on CA2 neurons against apoptosis induced by lipopolysaccharides (LPSs) [136]. In addition, a TCM compound known as NaoTaiFang has been shown to protect CA2 neurons following cerebral ischemia by increasing the expression of ferroportin (Fpn) and facilitating neuronal iron efflux [137]. Although studies have reported potential benefits of certain TCMs in the context of PD (for comprehensive reviews, refer to [138,139]), it remains unclear whether these TCMs effectively alleviate non-motor symptoms, particularly social disorders. Furthermore, it is necessary to determine whether identified TCMs have an impact on CA2 and the associated social cognitive dysfunction in PD.

**Table 3:** Medications and their effects on the CA2 region.

Medicine	Indication/Use	Effect on CA2 Region	Refs.
**Allopathic Medicines**			
Vasopressin	Diabetes insipidus; cardiac arrest	LTP inhibition in EC-CA2	[130]
Dantrolene	MH	Protection of ECS-induced apoptosis in CA2	[131]
Ketamine	Anesthetics	Protect apoptosis induced by ECS in CA2	[131]
Caffeine	N/A	Enhancement of synaptic transmission in CA2	[39]
L-DOPA	PD	Restoration of CA2 volume in PD patients	[134]
Fluoxetine	Depression; OCD	Reduction in synaptic protein and GR expression in CA2	[118]
Haloperidol	Schizophrenia; TS	Decrease in EAAT2 and NMDAR in the CA2	[132,133]
Clozapine	Schizophrenia	Decrease in EAAT2 and NMDAR in the CA2	[132,133]
Olanzapine	Schizophrenia	Decrease in NMDAR in the CA2	[133]
Oxytocin	Delivery medication; autism	Rescue of social impairment in an autism model in association with SST neurons in CA2	[41]
**Traditional Chinese Medicines (TCMs)**			
CS 4-O-sulfation	N/A	Increase in PNNs and excitatory–inhibitory synapses in CA2	[135]
NaoTaiFang	Activating blood and dissolving stasis	Protection of the CA2 neuronal population in cerebral ischemia	[137]
Dihydroartemisinin (extract from artemisinin)	Malaria	Protection against LPS-induced apoptosis in CA2	[136]
For more information on specific herbs and TCM formulas for treating PD symptoms, please refer to the following reviews:			[138,139]

LTP: long-term potentiation; ECS: electroconvulsive seizures; CS 4-O-sulfation: chondroitin 4-O-sulfation; PNNs: perineuronal nets; GR: glucocorticoid receptor; EAAT2: excitatory amino acid transporters; NMDAR: N-methyl-D-aspartate receptors; SST: somatostatin interneurons; LPS: lipopolysaccharide; MH: malignant hyperthermia; OCD: obsessive–compulsive disorder; TS: Tourette syndrome.

## 6. Summary of the Role of CA2 in Non-Motor Symptoms of Parkinson’s Disease

To summarize the putative role of CA2 in the non-motor symptoms of PD, it is important to recognize that although PD is primarily known for its motor symptoms, cognitive impairment and emotional dysregulation are also prevalent. Animal studies have demonstrated that PD-like mice exhibited impaired performance in social behavioral tests, which may indicate some involvement of the CA2 region in social interaction deficits. Furthermore, postmortem studies have revealed CA2 neuronal loss in PD patients, suggesting the vulnerability of this region to neurodegenerative processes. These findings suggest that the CA2 region may play a role in the non-motor symptoms of PD, particularly in social cognition.

## Figures and Tables

**Table 1:** Proteins that are highly expressed in CA2 neurons and their functions.

Name	Function	References
PCP4	Identification of the DG and CA2 regions	[12,15]
RGS14	Restriction of CA2 synaptic plasticity	[15,16,18]
STEP	LTP inhibition at EC-CA2 synapses	[15]
A1R	LTD enhancement at SC-CA2 synapses	[39,40]
AVPR1B	Enhancement of synaptic potentiation at SC-CA2 synapses Facilitation of social behavior	[21,24,26]
OXTR	Enhancement of synaptic potentiation at SC-CA2 synapses Facilitation of social behavior	[26,41]
MRs;	Facilitation of CA2-dependent behaviors	[38]
group III mGluRs;	Restriction of CA2 synaptic plasticity	[42]
cholinergic receptors	Induction of LTD at SC and EC CA2 synapses	[43,44]
**Related to PD**		
Substance P	Induction of SC and EC-CA2 synaptic plasticity	[31]
**α-synuclein**	Controversial	[45,46,47,48,49,50,51,52,53,54]

PCP4: Purkinje cell protein 4; RGS14: regulator of G protein signaling 14 protein; STEP: striatum-enriched protein–tyrosine phosphatase; A1R: A1 adenosine receptor; AVPR1B: vasopressin 1b receptor; OXTR: oxytocin receptor; MRs: mineralocorticoid receptors; Group III mGluRs: Group III metabotropic glutamate receptors.

**Table 2:** Comparison of rodent and human studies linking molecular targets and hippocampal formation to social behavior.

Social Symptoms	Related Molecules or Factors	Species	References
**Social Behavior and CA2**			
Social recognition memory	OXT/OXTR	Mouse	[25,41,107]
	Ageing	Mouse	[108]
	AVPR1B	Mouse	[24]
	CA2 pyramidal neurons	Mouse	[22]
	Juvenile stress	Rat; mouse	[109,110]
	High-frequency oscillations in CA2 neurons	Mouse	[111]
	PV interneurons and PNN	Mouse	[112,113,114]
Social novelty recognition	SuM-CA2 synapse	Mouse	[29]
Social aggression	AVPR1B	Mouse	[20,26]
	CA2-LS	Mouse	[20]
Social motivation	AVPR1B	Mouse	[115]
Social discrimination	LEC-CA2	Mouse	[116]
	Shank3B	Mouse	[117]
Sociability and social interaction	Juvenile stress	Rat	[109]
	Perinatal fluoxetine	Mouse	[118]
Social cognition	Schizophrenia patients; CA2 PV+ interneurons	Human; mouse	[119]
**Human Hippocampus and Social Behavior**			
Social recognition	Healthy people	Human	[104,105]
Social function	Connectivity of hippocampus in NVLD patients	Human	[106]
Face perception	Healthy people	Human	[103]
Social memory: familiar face and name recognition deficits	Case study	Human	[102]
**Human Social Behavior and CA2-Related Molecular Targets**			
Social stress and anxiety; social cognition and social approach; social behavior	Oxytocin; vasopressin	Human	[99,100]
Social anxiety; social discrimination; social behavior; social memory	RGS14	Human	[101]

OXT: oxytocin; OXTR: oxytocin receptor; AVPR1B: vasopressin 1b receptor; PV: parvalbumin; PNN: perineuronal net; SuM: supramammillary nuclei; LS: lateral septum; LEC: lateral entorhinal cortex; NVLD: nonverbal learning disabilities; RGS14: regulator of G protein signaling 14 protein.

## Data Availability

Not applicable.
